# Disjunct habitat of cryptic *Terebellides* (Annelida, Trichobranchidae) species shows a phylogenetic link between polychaetes from the White and the North Seas

**DOI:** 10.1038/s41598-023-49785-9

**Published:** 2023-12-21

**Authors:** A. A. Kudryavtseva, U. S. Novoyatlova, A. Chuyko, D. R. Gaeva, A. V. Vlasov, I. V. Manukhov

**Affiliations:** 1 Moscow Institute of Physics and Technology, 141701 Dolgoprudny, Russia; 2 Laboratory of Microbiology, BIOTECH University, 125080 Moscow, Russia; 3 Shirshov Institute of Oceanology, Russian Academy of Sciences, Moscow, Russia; 4 Joint Institute for Nuclear Research, 141980 Dubna, Russia

**Keywords:** Biogeography, Population genetics, Climate-change ecology

## Abstract

Understanding the distribution and biodiversity of marine species is crucial for developing effective conservation strategies and maintaining the health of global ecosystems. Advancements in molecular data utilization have significantly improved our understanding of biodiversity within the genus *Terebellides*. In this study, we conducted a phylogenetic analysis on polychaete samples from the Kandalaksha Bay, White Sea, revealing their affiliation with a putative undescribed species of the genus *Terebellides* found in two locations of the North Sea. Interestingly, this species was not detected in the Norwegian and Barents Seas, leading us to propose a disjunct distribution scenario for this *Terebellides* species. This unique distribution pattern might be attributed to the succession of polychaetes by new species, facilitated by the Gulf Stream and a climate change role in driving shifts in species' ranges and altering marine ecosystem dynamics.

## Introduction

The genus *Terebellides* Sars, 1835 (Annelida, Trichobranchidae) and its first representative, *Terebellides stroemi* Sars, 1835^1^, belonging to the class Polychaeta (bristle worms), was discovered in Norway in 1835 (Sars M). This species prefers soft sediments and inhabits a wide range of depths. For a long time, *T. stroemi* was considered a cosmopolitan species, with its representatives being widely distributed in the Arctic Ocean, Barents, Okhotsk Seas, but also reported from the Atlantic coasts of North America, Europe and Africa, and from the Mediterranean and Black seas^2,3^. According to WoRMS (World Register of Marine Species, https://www.marinespecies.org/), there are currently a total of 86 accepted species belonging to the genus *Terebellides*. However, subsequent research has significantly altered the understanding of the species diversity hidden within the representatives of this genus in European waters^4–8^. These authors greatly increased the number of described species in the North-East Atlantic (NEA), and highlighted the existence of several cryptic species hidden among known *Terebellides*^5,6^. The phylogenetic analysis performed by Nygren and colleagues^5^, based on the sequences of four genes (CO1, 16S, ITS, and 28S) of over five hundred samples, revealed the presence of twenty-eight separate clades within the *Terebellides* from NEA, corresponding to different species (*T. stroemi*, *T. gracilis* Malm, 1874, *T. atlantis* Williams, 1984, *T. williamsae* Jirkov, 1989, *T. irinae* Gagaev, 2009, *T. bigeniculatus* Parapar, Moreira & Helgason, 2011 and *T. shetlandica* Parapar, Moreira & O'Reilly, 2016), with the remaining clades belonging to new, yet undescribed molecular lineages.

In our study, we conducted a multilocus phylogenetic analysis of the sequences (CO1, 16S, ITS, and 28S) resulting from six *Terebellides* specimens collected at the Chernorechenskaya Inlet in Kandalaksha Bay on the White Sea to assign them to putative species within the NEA group according to the work of Nygren and colleagues^5^.

## Materials and methods

### Specimens and study area

Six *Terebellides* specimens were collected near Olenevsky Island at Chernorechenskaya Inlet in Kandalaksha Bay on the White Sea (66.52064º, 33.113101º). The polychaetes were collected using a dredge on the ship "S. Dezhnev" of the Youth Educational Expeditions (http://expeditions.ru) in July 2021 and July 2023. The polychaetes were relaxed in a 50 mM MgSO_4_ solution and observed under a light microscope. Subsequently, the specimens were transferred to a preservation buffer (100 mM EDTA, 100 mM Tris–HCl, 150 mM NaCl) and dissected to separate the intestine, gonads, and muscle tissue. DNA was extracted from tissues of six specimens (listed in Table S1). Also, two specimens belonging to the species *Polycirrus medusa* Grube, 1850 (family Terebellidae) were collected in the same region (Table S1), which sequences intended to be used as an outgroup.

### DNA extractions, PCR, and sequencing

DNA extraction followed the method described in Sidoruk et al*.*^9^, with preliminary grinding of samples in liquid nitrogen or STE stabilizing buffer^10^. Tissue from the pygidium (parts of pygidium) and seminal vesicles were used for DNA extraction. The extracted DNA was used as a template to amplify fragments of mitochondrial cytochrome oxidase I (COI) and ribosomal RNA (16S) genes, and nuclear sequences of the non-transcribed spacer region (ITS2) and nuclear ribosomal RNA (28S). Primers and PCR conditions are shown in Table 1. All PCR reactions were made using ScreenMix PCR mix (Evrogen, Russia) according to the manual. Products were purified using CleanUp mini set (Evrogen, Russia).

**Table 1:** Oligonucleotides used for DNA amplification mitochondrial cytochrome oxidase I (COI) and ribosomal RNA (16S) genes, and nuclear sequences of the non-transcribed spacer region (ITS2) and nuclear ribosomal RNA (28S).

Gene	Primers	Primer melting temperature, ºC
COI	LCO1490 (GGTCAACAAATCATAAAGATATTGG HCO2198 (TAAACTTCAGGGTGACCAAAAAATCA)	50
16S	16SANNF (GCGGTATCCTGACCGTRCWAAGGTA) or 16SARL (CGCCTGTTTATCAAAAACAT) 16SBRH (CCGGTCTGAACTCAGATCACGT)	50
ITS2	ITS58SF (GAATTGCAGGACACATTGAAC) ITS28SR (ATGCTTAAATTCAGCGGGT)	58
28S	28SC1 (ACCCGCTGAATTTAAGCAT) 28SD2 (TCCGTGTTTCAAGACGG)	58

PCR amplicons were sequenced using the Sanger method at Evrogen JSC (Russia) in both directions with the primers used for fragment amplification.

### Sequence analysis

A manual examination of sequence chromatograms was carried out in SnapGene 2.3.2 (GSL Biotech LLC). Comparisons of individual sequences and similarity analysis were done using BLAST (http://blast.ncbi.nlm.nih.gov/)^11^.

Multiple sequence alignment was performed with MEGA-X software (https://www.megasoftware.net/)^12^ using ClustalW and then MUSCLE algorithms. Five datasets were tested: COI, 16S, 28S, ITS and a concatenated dataset. We used ModelFinder (http://www.iqtree.org/ModelFinder/)^13^ to choose the best evolutionary model for our alignments. We used the Akaike information criteria (AICc) to select the best-fit substitution model for each dataset (best-fit models are listed in Table S2). The COI gene dataset was divided into two partitions by base positions (1st, 2nd *vs* 3rd).

Phylogenetic relationships among North-East Atlantic species of the *Terebellides* genus were examined using the Bayesian analysis (BA) performed with MrBayes (ver. 3.2.7, see https://nbisweden.github.io/MrBayes/index.html) program^14^. Two runs of 10,000,000 generations with four chains (one cold and three heated) were performed. Chains were sampled every 10,000 generations. The average value of the Potential Scale Reduction Factor (PSRF) was 1.002 and the average standard deviation of split frequencies was 0.01123 at the end of the analysis, indicating convergence was achieved and a good sample from the posterior probability (PP) distribution was obtained. The resulting trees’ effective sampling sizes (ESS) were more than 200 and were calculated using Tracer software (ver. 1.7, see https://beast.community/tracer)^15^.

To construct the trees, we used sequences of four genes (COI, 16S, 28S, ITS) of six specimens we obtained and two *Polycirrus medusa* specimens (used as an outgroup). Two specimens from the study of Nygren et al. for each clade were used as the references (Table S1 shows all the data used for tree constructing)^5^. The uncorrected genetic distances were calculated in MEGA-X for the concatenated dataset of four genes. Tree visualization was performed with Tree Of Life (iTOL) v5 online tool (https://itol.embl.de)^16^.

### Data analysis

A one tailed t-test was performed to verify statistical significance of the differences in Clade 4 (as well as Clade 12) distribution between locations.

## Results

**Figure 1:**
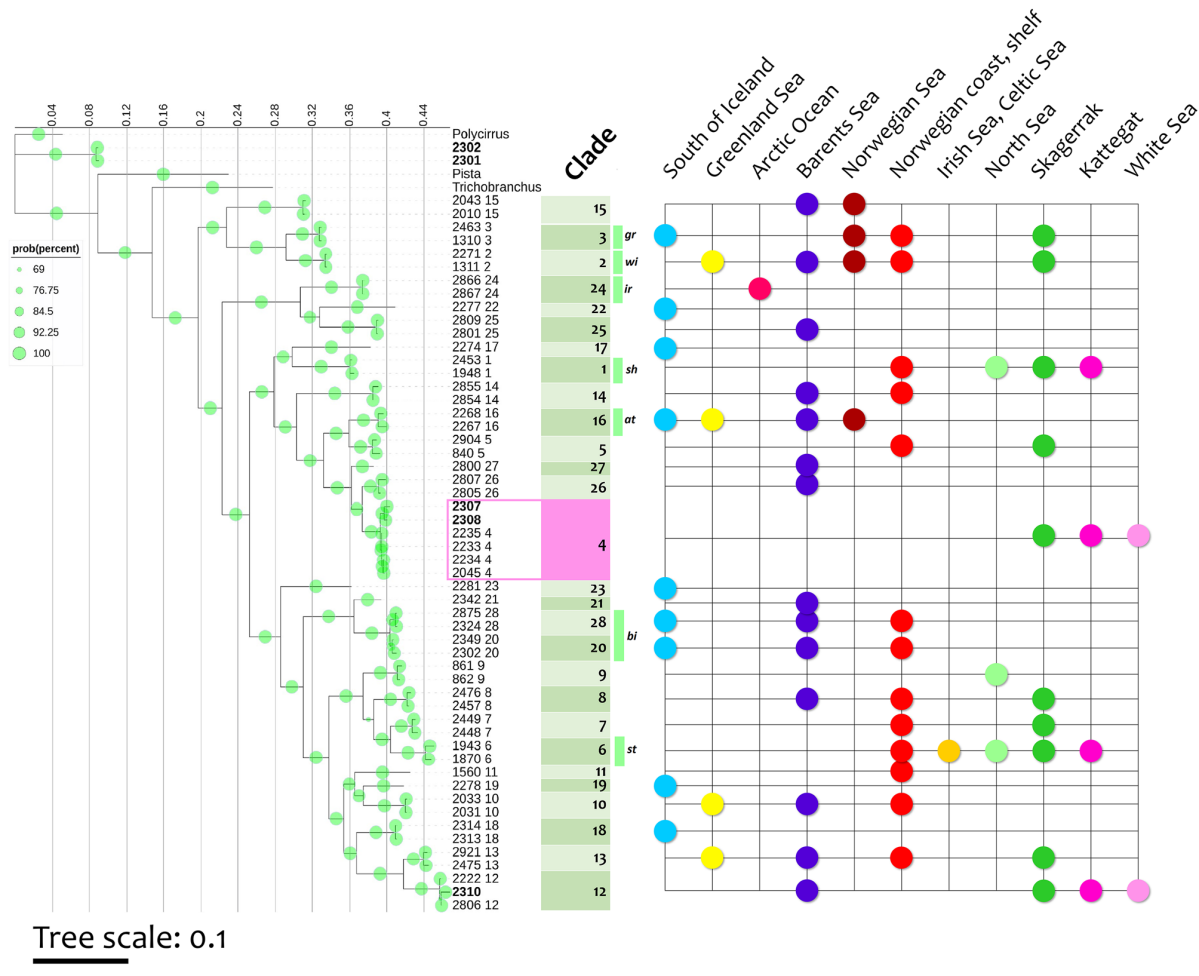
Phylogenetic tree based on the analysis of combined sequences of four fragments: mitochondrial (COI, 16S rDNA) and nuclear (ITS2, 28S rDNA), totaling approximately 2574/2474 aligned positions, constructed using the BA method. The sequences, represented by numbers, were retrieved from Nygren et al. study^5^, and some were obtained in the present study (bold numbers). Clade numbers are from Nygren et al. study^5^. Clades 4 and 12 refers to undescribed species in the study by Nygren and colleagues, previously not reported in the White Sea. The nodes are marked with Bayesian posterior probabilities (PP). Biogeographical regions where the specimens of each clade were collected are indicated by color circles: Kattegat (purple), Skagerrak (dark green), North Sea (light green), Irish Sea and Celtic Sea (orange), Norwegian coast and shelf (red), Norwegian Sea (brown), Barents Sea (dark blue), Arctic Ocean (pink-red), Greenland Sea (yellow), South Iceland (light blue) – marked according to the study by Nygren and colleagues^5^; White Sea (light pink) indicate the geographical region for the samples 2307, 2308 and 2310, collected in the present study. Formally described species are shown according to the Nygren and colleagues^5^ *Terebellides irinae* (ir), *T. atlantis* (at), *T. bigeniculatus* (bi), *and T. shetlandica* (sh).

Phylogenetic trees for each gene (COI, ITS2, 28S, and 16S) individually and as a concatenated dataset, as described by Nygren and colleagues^5^, were constructed using the Bayesian analyses (BA) method (Figs. 1, S1–S4). It turned out that chosen models and two sequences (specimens) for every clade were sufficient to reproduce the topology of the phylogenetic tree for NEA *Terebellides* obtained by Nygren and collegues^5^.

**Figure 2:**
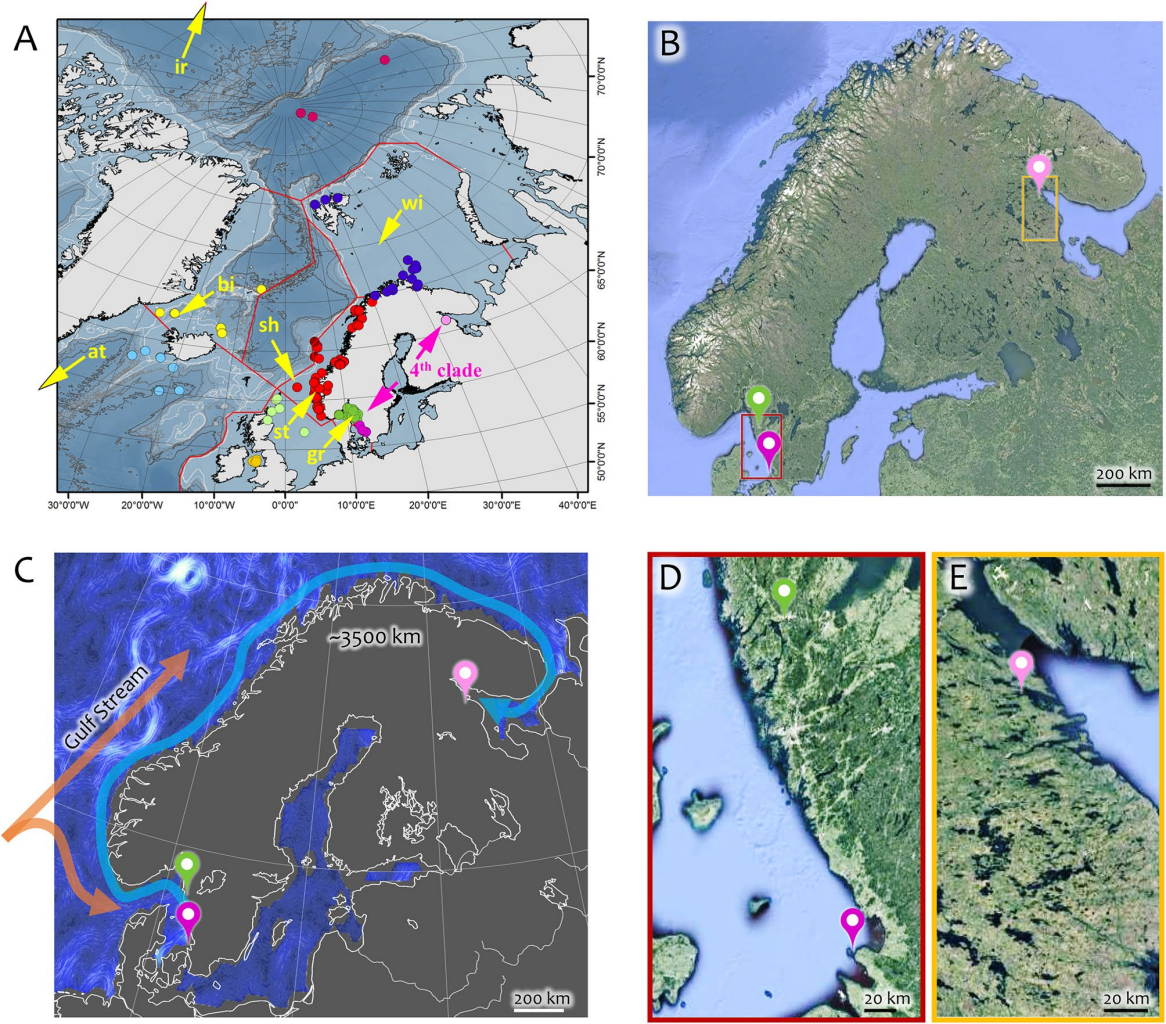
(**A**) Sample collection sites and biogeographical regions and type localities of *Terebellides irinae* (ir), *T. atlantis* (at), *T. bigeniculatus* (bi), and *T. shetlandica* (sh) from the study by Nygren and colleagues^5^ (indicated by yellow arrows). Type localities for *T. irinae* and *T. atlantis* are located outside the map area. Biogeographical regions of sample collection sites are denoted by colors: Kattegat (purple), Skagerrak (dark green), North Sea (light green), Irish Sea and Celtic Sea (orange), Norwegian coast and shelf (red), Norwegian Sea (brown), Barents Sea (dark blue), Arctic Ocean (pink-red), Greenland Sea (yellow), South Iceland (light blue). The sample collection sites for the Clade 4 are shown with pink geotags, including the samples 2307, 2308, 2311 and 2312 collected in the Kandalaksha Bay of the White Sea. (**B**) Sample collection sites in Kandalaksha Bay of the White Sea and Kattegat. (**C**) representation of the ocean currents (https://earth.nullschool.net/#current/ocean/surface/currents), and the distance between possible disjunct habitats of *Terebellides* species belonging to Clade 4. Detailed map of sample collection sites in: (**D**) Kattegat and (**E**) Kandalaksha Bay of the White Sea. (**A**) is reprinted from the study by Nygren and colleagues^5^ with the permissions from the Public Library of Science (PLOS)**.** Maps on the (**B**,**D**,**E**) are built using Google Maps (2023).

The data in Fig. 1 shows that the specimens 2307 and 2308 obtained in the present study cluster with samples 2045 4, 2233 4, 2234 4 and 2235 4, which are ascribed to Clade 4 in the study by Nygren and colleagues^5^ and considered to be a different undescribed species. Moreover, phylogenetic trees built for each of the four genes’ datasets separately (Figs. S1–4) also showed that the studied specimens from the White Sea belong to Clade 4. In addition to 2307 and 2308, the 28S (Fig. S3) and ITS (Fig. S4) trees also include in Clade 4 the *Terebellides* specimens 2311 and 2312 collected in 2023 in the Kandalaksha Bay (Table S1). Representatives of Clade 4 were found by Nygren and colleagues^5^ only within two locations in the North Sea and were not encountered all across the Norwegian coast (Fig. 2). The samples 2307 and 2308 form their own separate cluster within Clade 4, which may be explained by some genetic isolation of the White Sea and the North Sea populations of Polychaeta (Fig. 1).

To test the distances between the species sequences we tested two more neighboring Clades 26 and 27 (also corresponding to undescribed species) which are the closest genetically to Clade 4 according to Nygren and colleagues^5^. Uncorrected distances were calculated and analyzed (Table S3). Intragroup distances between representatives from Clade 4 (including 2307, 2308, 2311 and 2312) did not exceed the distances between Clade 4 and genetically neighboring Clade 26 or Clade 27.

We also found *Terebellides* which we classified to Clade 12 [specimen 2310 (Figs. 1, S1–4) and 2309 (Figs. S2–S4)], described in the study by Nygren and collegues^5^. Representatives of Clade 12 in addition to White Sea, Kattegat and Skagerrak are also present in the Barents Sea (Fig. 1).

Note that *Terebellides* often live sympatrically, occupying one habitat with several species, but among them there are examples of non-sympatric residence. Thus, according to Fig. 1, Clades 24 and 6 live separately in their niches (the Arctic Ocean and the Irish Sea, respectively).

Figure 2A displays the sampling locations of specimens characterized by COI, ITS2, 28S, and 16S gene sequences currently available in the GenBank database. Representatives from Clade 4 are identified in both the North and White Seas, as shown in Fig. 2B within a red rectangle (Fig. 2D) and a yellow rectangle (Fig. 2E), respectively. The estimated distance for potential migration through coastal currents between these sampling sites is approximately 3500 km (Fig. 2C). Consequently, it is highly likely that various *Terebellides* species, including Clade 4, were initially distributed evenly along the coast between these sites by the Gulf Stream, before the Clade 4 population was replaced by other species.

## Discussion

Humanity wonders about the reasons for the disjunct distribution of different species from all branches of the tree of life^17–19^. The study of the distribution and genetic diversity of *Terebellides* species offers valuable insights into the biogeography and adaptation of these polychaetes. In this context, we find it particularly noteworthy that no specimens belonging to Clade 4 were found in the Norwegian Sea, despite their presence in the Kattegat/Skagerrak and White Sea. A disjunct distribution of Clade 4 was confirmed by a one-tailed t-test (Tables S4, S5). The data obtained show that the probability of the different Clade 4 distribution in the Kattegat/Skagerrak and White Sea compared to the Norwegian coast and shelf is above than 99.9%.

This intriguing pattern may be attributable to the succession of polychaetes in the Norwegian Sea by new species introduced by the Gulf Stream. The ability of different *Terebellides* species to coexist within the same habitat indicates the possibility of sympatric speciation. Despite this, interspecific competition apparently occurs due to the limitation of favorite habitats and, as a result, the emergence of preferential habitat of specific species in different areas.

It should be noted that in Kandalaksha Bay we also found *Terebellides* which we classified to Clade 12 (specimens 2309 (Figs. 1, S1–4) and 2310 (Figs. S2–S4), described in the Nygren et al.^5^ study. However, Clade 12 is also present in the Barents Sea (Fig. 1) so the range gap is not so impressive. But the presence of this gap on the Norwegian west coast suggests that Clade 12 is exposed to the same processes as Clade 4. The probability of the identical Clade 12 distribution in the Kattegat/Skagerrak, Barents and White Seas compared to the Norwegian coast and shelf is also below 0.0001 (Table S6).

The Clade 4 preservation within Kattegat and Skagerrak or Kandalaksha Bay may be associated with both the distance from the Gulf Stream and other unique features of the ecological niches occupied by different polychaetes. For instance, the Kattegat and Skagerrak straits in the North Sea and the Chernorechenskaya Inlet at the Kandalaksha Bay in the White Sea might share similarities in their periodic salinity fluctuations^20,21^. These fluctuations are determined by tidal currents at the boundary with the Baltic Sea in the first case, and by tidal currents of the White Sea, which also exhibits low salinity, in the second case.

It is worth noting that Skagerrak appears to be a more open water area than Kattegat strait or Kandalaksha Bay. According to a one-tailed t-test the differences between the Clade 4 distribution in Skagerrak and the Norwegian coast and shelf are not so great: p-value around 0.2 (Table S5). It seems that Clade 4 representatives prefer shallow, closed, slightly salted reservoirs.

In the current discourse on the origins of disjunct distributions in aquatic animals, four predominant hypotheses are articulated^23,24^. According to one of these hypotheses, epiplanktonic larval dispersal creates conditions for the disjunct distribution. Larval transport could lead to the introduction of new species along the Norwegian coast, followed by a succession process. However, the ability of *Terebellides* to exhibit sympatric coexistence among different species within a single range appears to counter the potential succession by new species within the Norwegian shelf Clades 4 and 12. Another consideration is the larval washout from the Norwegian shelf by currents, potentially resulting in a gradual decline in species abundance in the region and the accumulation of populations in the semi-enclosed basins of the White and Baltic Seas.

The potential for *Terebellides* larvae to be transported by shipping is also theoretically plausible. While the most direct route from the Baltic to the White Sea is via the White Sea-Baltic Channel, this passage entails prolonged exposure to freshwater conditions, which is detrimental to larval survival. An alternative migration path around the Scandinavian peninsula would have presumably led to a broader dispersal of Clade 4 individuals along the coast.

While Clades 4 and 12 predominantly inhabit different depths as detailed in Table S4, both exhibit a disjunct distribution on the Norwegian shelf. This pattern reinforces our hypothesis regarding the influence of the Gulf Stream on the formation of disjunct distributions in the North Atlantic.

There are reports of the influence of the Gulf Stream on speciation, as demonstrated by the separation of American and European eels following the closure of the Isthmus of Panama and the subsequent intensification of marine currents^25^. The role of the Gulf Stream in facilitating the dispersal of species reflected in the biogeography of fish and corals is shown in several works^26,27^. Temperature and salinity fluctuations^22^, alongside the long-distance dispersal of larvae by currents, may influence on polychaetes speciation. Our study introduces the novel proposition that the Gulf Stream contributes to the emergence of disjunct distributions in polychaetes.

Further investigation into the factors contributing to the disjunct distribution of *Terebellides* species belonging to Clade 4 is crucial for understanding the underlying mechanisms of their biogeography. As these polychaetes play a significant role in benthic ecosystems, their distribution and abundance may have broader implications for community structure, nutrient cycling, and overall ecosystem functioning. Hence, it is essential to explore the environmental variables, ecological interactions, and historical factors that shape the observed distribution patterns.

It is also worth considering the potential impact of climate change on the distribution and range shifts of these species^28,29^. As ocean temperatures continue to rise, the distribution of various marine species may change, either through range expansions, contractions, or shifts in response to changing environmental conditions. Consequently, the disjunct distribution observed in Clade 4 may be influenced by changing oceanic conditions, which could further modify the structure of marine communities and impact ecosystem services.

Future research should focus on a more extensive sampling effort across the entire distribution range of *Terebellides* species to better understand the factors contributing to their disjunct distribution. Additionally, utilizing advanced genomic approaches and modeling techniques could provide insights into the population structure, gene flow, and historical demographic events that may have shaped the distribution of these organisms.

## Final remarks

The phylogenetic analysis of the ITS, COI, 28S, and 16S gene sequences of the *Terebellides* specimens 2307, 2308, 2311 and 2312 from Chernorechenskaya Inlet at Kandalaksha Bay in the White Sea reveals that the studied specimens cluster with *Terebellides* that were previously known from the Kattegat and Skagerrak straits on the North Sea. The lack of related worms among more than 500 samples in the Norwegian and Barents Seas supports the hypothesis of a disjunct distribution for Clade 4 *Terebellides*. This study highlights the complex biogeographic patterns of these marine organisms, shedding light on the processes that drive their distribution and adaptation in various marine environments. In conclusion, this study on the distribution and genetic diversity of *Terebellides* species underscores the importance of investigating the complex biogeographic patterns of marine organisms. The disjunct distribution of species within Clade 4 offers a unique opportunity to explore the ecological and evolutionary factors influencing the distribution and adaptation of these polychaetes. A comprehensive understanding of these factors will help inform conservation efforts and enhance our knowledge of marine ecosystem dynamics.

### Supplementary Information

Supplementary Information.

## Data Availability

The sequences generated during the current study are available in the GenBank repository (Table S1). The extracted DNA is stored in the Molecular Genetics Laboratory, MIPT. Clade 4 and Clade 12 specimens, as well as *Polycirrus medusa* collected specimens are stored in the museum of Shirshov Institute of Oceanology, Russian Academy of Sciences (INV0002301-INV0002312).

## References

[1] Sars, M. *Beskrivelser og iagttagelser over nogle mærkelige eller nye i havet ved den bergenske kyst levende dyr af polypernes, acalephernes, radiaternes, annelidernes, og molluskernes classer: med en kort oversigt over de hidtil af forfatteren sammesteds fundne ar*. (Dahl, 1835).

[2] Oug E, Bakken T, Kongsrud JA (2014). Original specimens and type localities of early described polychaete species (Annelida) from Norway, with particular attention to species described by O.F. Müller and M. Sars. Museum Victoria.

[3] Hutchings P, Kupriyanova E (2018). Cosmopolitan polychaetes—Fact or fiction? Personal and historical perspectives. Invertebr. Syst..

[4] Parapar J, Martin D, Moreira J (2020). On the diversity of Terebellides (Annelida, Trichobranchidae) in West Africa, seven new species and the redescription of *T. africana* Augener, 1918 stat. prom. Zootaxa.

[5] Nygren A (2018). A mega-cryptic species complex hidden among one of the most common annelids in the north east Atlantic. PLoS ONE.

[6] Parapar J, Capa M, Nygren A, Moreira J (2020). To name but a few: Descriptions of five new species of terebellides (annelida, trichobranchidae) from the north east atlantic. Zookeys.

[7] Lavesque N, Hutchings P, Daffe G, Nygren A, Londoño-Mesa MH (2019). A revision of the French Trichobranchidae (Polychaeta), with descriptions of nine new species. Zootaxa.

[8] Barroso M, Moreira J, Capa M, Nygren A, Parapar J (2022). A further step towards the characterisation of *Terebellides* (Annelida, Trichobranchidae) diversity in the Northeast Atlantic, with the description of a new species. Zookeys.

[9] Sidoruk KV, Levitin EI, Sviridov BV, Piksasova OV, Shustikova TE (2021). A method of DNA extraction from a wide range of objects via treatment with ammonium salts. Appl. Biochem. Microbiol..

[10] Sambrook, J. & Russell, D. W. *Molecular Cloning: A Laboratory Manual (3-volume set)*. (2001).

[11] Johnson M (2008). NCBI BLAST: A better web interface. Nucleic Acids Res..

[12] Kumar S, Stecher G, Li M, Knyaz C, Tamura K (2018). MEGA X: Molecular evolutionary genetics analysis across computing platforms. Mol. Biol. Evol..

[13] Kalyaanamoorthy S, Minh BQ, Wong TKF, Von Haeseler A, Jermiin LS (2017). ModelFinder: Fast model selection for accurate phylogenetic estimates. Nat. Methods.

[14] Ronquist F (2012). Mrbayes 3.2: Efficient Bayesian phylogenetic inference and model choice across a large model space. Syst. Biol..

[15] Rambaut A, Drummond AJ, Xie D, Baele G, Suchard MA (2018). Posterior summarization in Bayesian phylogenetics using Tracer 1.7. Syst. Biol..

[16] Letunic I, Bork P (2021). Interactive tree of life (iTOL) v5: An online tool for phylogenetic tree display and annotation. Nucleic Acids Res..

[17] Dalui S (2021). Geological and Pleistocene glaciations explain the demography and disjunct distribution of red panda (*A. fulgens*) in eastern Himalayas. Sci. Rep..

[18] Feng B (2016). Multilocus phylogenetic analyses reveal unexpected abundant diversity and significant disjunct distribution pattern of the Hedgehog Mushrooms (*Hydnum* L.). Sci. Rep..

[19] Chao CT, Kuo CC, Chang JT, Chai MW, Liao PC (2021). Evolution of floral characters and biogeography of Heloniadeae (Melanthiaceae): An example of breeding system shifts with inflorescence change. Sci. Rep..

[20] Stigebrandt A, Gustafsson BG (2003). Response of the Baltic Sea to climate change—Theory and observations. J. Sea Res..

[21] Dolotov YS (2005). Monitoring tidal conditions in estuaries of the Karelian coast of the White Sea. Water Resour..

[22] Silver A, Gangopadhyay A, Gawarkiewicz G, Fratantoni P, Clark J (2023). Increased gulf stream warm core ring formations contributes to an observed increase in salinity maximum intrusions on the Northeast Shelf. Sci. Rep..

[23] Castilla JC, Guiñez R (2000). Disjoint geographical distribution of intertidal and nearshore benthic invertebrates in the Southern Hemisphere. Rev. Chil. Hist. Nat..

[24] Haydar D (2012). What is natural? The scale of cryptogenesis in the North Atlantic Ocean. Divers. Distrib..

[25] Jacobsen MW (2014). Speciation and demographic history of Atlantic eels (*Anguilla anguilla* and *A. rostrata*) revealed by mitogenome sequencing. Heredity.

[26] Guzmán-Méndez IA (2017). First genetically confirmed record of the invasive devil firefish Pterois miles (Bennett, 1828) in the Mexican Caribbean. BioInvasions Rec..

[27] Hoeksema BW, Roos PJ, Cadée GC (2012). Trans-Atlantic rafting by the brooding reef coral Favia fragum on man-made flotsam. Mar. Ecol. Prog. Ser..

[28] Thuiller W (2004). Patterns and uncertainties of species’ range shifts under climate change. Glob. Chang. Biol..

[29] Burrows MT (2014). Geographical limits to species-range shifts are suggested by climate velocity. Nature.

