# Supplementary material for: Disjunct habitat of cryptic Terebellides (Annelida, Trichobranchidae) species shows a phylogenetic link between polychaetes from the White and the North Seas
Source: Sci Rep. 2023 Dec 21;13:22926. doi: 10.1038/s41598-023-49785-9 (PMC10739723; doi:10.1038/s41598-023-49785-9)
Supplement: Supplementary file 1 — Supplementary Information. [file 41598_2023_49785_MOESM1_ESM.docx]

**Table S1**. Specimens and sequences used for the analysis.

| Specimen | COI | 16S | 28S | ITS | Source | GenBank accession numbers |
| --- | --- | --- | --- | --- | --- | --- |
| Ter_white*  (INV0002308)** | + | + | + | + | July 2022, this study | Ter_white_COI: OQ925867  Ter_white_16S: OQ925892  Ter_white_ITS: OQ929935  Ter_white_28S: OQ924991 |
| Ter_pink  (INV0002307) | + | + | + | + | July 2022, this study | Ter_pink_CO: OQ925868  Ter_pink_16S: OQ925893  Ter_pink_ITS: OQ929936  Ter_pink_28S: OQ924992 |
| Red1  *(Polycirrus medusa)*  (INV0002301) | + | + | + | + | July 2023, this study | Red1_COI: OR473702  Red1_16S: OR482380  Red1_28S: OR473709  Red1_ITS: OR482386 |
| Red2  *(Polycirrus medusa)*  (INV0002302) | + | + | + | + | July 2023, this study | Red1_COI: OR473703  Red1_16S: OR482381  Red1_28S: OR473710  Red1_ITS: OR482387 |
| Pink2  (INV0002309) | + | + | + | + | July 2023, this study | Pink2_COI: OR554227  Pink2_16S: OR546347  Pink2_28S: OR554010  Pink2_ITS: OR552465 |
| Pink3  (INV0002310) | + | + |  | + | July 2023, this study | Pink3_COI: OR554228  Pink3_16S: OR546348  Pink3_ITS: OR552466 |
| Yellow2  (INV0002311) |  |  | + | + | July 2023, this study | Yellow2_28S: OR554011  Yellow2_ITS: OR552467 |
| Yellow3  (INV0002312) |  | + | + | + | July 2023, this study | Yellow3_16S: OR546349  Yellow3_28S: OR554012  Yellow3_ITS: OR552468 |
| *Polycirrus* | + | + | + | - | Downloaded from GenBank | COI: JX423769  16S rDNA: JX423681  28S rDNA: JN936481 |
| *Pista* | + | + | + | - | Downloaded from GenBank | COI: EU239688  16S rDNA: NC011011  28S rDNA: DQ790057 |
| *Trichobranchus*  (Trichobranchus roseus) | + | + | + | - | Downloaded from GenBank | COI: MH113923  16S rDNA: MG025442 |
| 1948_1 | + | + | + | + | Nygren et al. study | COI: MG024931  16S: MG025443  28S: MG024522  ITS: MG025357 |
| 2453_1 | + | + | + | + | Nygren et al. study | COI: MG024943  16S: MG025445  28S: MG024539  ITS: MG025360 |
| 1311_2 | + | + | + | + | Nygren et al. study | COI: MG024959  16S: MG025446  28S: MG024556  ITS: MG025361 |
| 2271_2 | + | + | + | + | Nygren et al. study | COI: MG024967  16S: MG025447  28S: MG024562  ITS: MG025362 |
| 1310_3 | + | + | + | + | Nygren et al. study | COI: MG024990  16S: MG025449  28S: MG024584  ITS: MG025366 |
| 2463_3 | + | + | + | + | Nygren et al. study | COI: MG024995  16S: MG025450  28S: MG024592  ITS: MG025367 |
| 2233_4 | + | + | + | + | Nygren et al. study | COI: MG025047  16S: MG025453  28S: MG024646  ITS: MG025371 |
| 2235_4 | + | + | + | + | Nygren et al. study | COI: MG025049  16S: MG025455  28S: MG024648  ITS: MG025373 |
| 2045_4 | + | + | + | + | Nygren et al. study | COI: MG025039  16S: MG025456  28S: MG024638  ITS: MG025370 |
| 2234_4 | + | + | + | + | Nygren et al. study | COI: MG025048  16S: MG025454  28S: MG024647  ITS: MG025372 |
| 840_5 | + | + | + | + | Nygren et al. study | COI: MG025053  16S: MG025457  28S: MG024651  ITS: MG025374 |
| 2904_5 | + | + | + | + | Nygren et al. study | COI: MG025064  16S: MG025460  28S: MG024662  ITS: MG025377 |
| 1870_6 | + | + | + | + | Nygren et al. study | COI: MG025090  16S: MG025461  28S: MG024683  ITS: MG025379 |
| 1943_6 | + | + | + | + | Nygren et al. study | COI: MG025097  16S: MG025462  28S: MG024690  ITS: MG025380 |
| 2448_7 | + | + | + | + | Nygren et al. study | COI: MG025110  16S: MG025464  28S: MG024697  ITS: MG025384 |
| 2449_7 | + | + | + | + | Nygren et al. study | COI: MG025111  16S: MG025465  28S: MG024698  ITS: MG025385 |
| 2457_8 | + | + | + | + | Nygren et al. study | COI: MG025148  16S: MG025467  28S: MG024720  ITS: MG025387 |
| 2476_8 | + | + | + | + | Nygren et al. study | COI: MG025149  16S: MG025468  28S: MG024721  ITS: MG025388 |
| 862_9 | + | + | + | + | Nygren et al. study | COI: MG025158  16S: MG025470  28S: MG024730  ITS: MG025391 |
| 861_9 | - | + | + | + | Nygren et al. study | 16S: MG025471  28S: MG024729  ITS: MG025390 |
| 2031_10 | + | + | + | + | Nygren et al. study | COI: MG025163  16S: MG025472  28S: MG024734  ITS: MG025392 |
| 2033_10 | + | + | + | + | Nygren et al. study | COI: MG025165  16S: MG025473  28S: MG024735  ITS: MG025393 |
| 1560_11 | + | + | + | + | Nygren et al. study | COI: MG025171  16S: MG025475  28S: MG024738  ITS: MG025395 |
| 2222_12 | + | + | + | + | Nygren et al. study | COI: MG025188  16S: MG025479  28S: MG024750  ITS: MG025400 |
| 2806_12 | + | + | + | + | Nygren et al. study | COI: MG025192  16S: MG025480  28S: MG024754  ITS: MG025401 |
| 2475_13 | + | + | + | + | Nygren et al. study | COI: MG025219  16S: MG025481  28S: MG024777  ITS: MG025407 |
| 2921_13 | + | + | + | + | Nygren et al. study | COI: MG025222  16S: MG025482  28S: MG024780  ITS: MG025408 |
| 2854_14 | + | + | + | + | Nygren et al. study | COI: MG025240  16S: MG025484  28S: MG024800  ITS: MG025411 |
| 2855_14 | + | + | + | + | Nygren et al. study | COI: MG025241  16S: MG025485  28S: MG024801  ITS: MG025412 |
| 2010_15 | + | + | + | + | Nygren et al. study | COI: MG025249  16S: MG025487  28S: MG024807  ITS: MG025414 |
| 2043_15 | + | + | + | + | Nygren et al. study | COI: MG025256  16S: MG025488  28S: MG024816  ITS: MG025416 |
| 2267_16 | + | + | + | + | Nygren et al. study | COI: MG025258  16S: MG025490  28S: MG024818  ITS: MG025417 |
| 2268_16 | + | + | + | + | Nygren et al. study | COI: MG025259  16S: MG025491  28S: MG024819  ITS: MG025418 |
| 2274_17 | + | + | + | + | Nygren et al. study | COI: MG025313  16S: MG025496  28S: MG024868  ITS: MG025423 |
| 2313_18 | + | + | + | + | Nygren et al. study | COI: MG025315  16S: MG025497  28S: MG024869  ITS: MG025424 |
| 2314_18 | + | + | + | + | Nygren et al. study | COI: MG025316  16S: MG025498  28S: MG024870  ITS: MG025425 |
| 2278_19 | + | + | + | + | Nygren et al. study | COI: MG025317  16S: MG025499  28S: MG024871  ITS: MG025426 |
| 2302_20 | + | + | + | + | Nygren et al. study | COI: MG025318  16S: MG025500  28S: MG024872  ITS: MG025427 |
| 2349_20 | + | + | + | + | Nygren et al. study | COI: MG025319  16S: MG025501  28S: MG024873  ITS: MG025428 |
| 2342_21 | + | + | + | + | Nygren et al. study | COI: MG025320  16S: MG025502  28S: MG024874  ITS: MG025429 |
| 2277_22 | + | + | + | + | Nygren et al. study | COI: MG025338  16S: MG025503  28S: MG024876  ITS: MG025430 |
| 2281_23 | + | - | + | + | Nygren et al. study | COI: MG025339  16S: MG025504  28S: MG024877  ITS: MG025431 |
| 2866_24 | + | + | + | + | Nygren et al. study | COI: MG025342  16S: MG025506  28S: MG024879  ITS: MG025432 |
| 2867_24 | + | + | + | + | Nygren et al. study | COI: MG025343  16S: MG025507  28S: MG024880  ITS: MG025433 |
| 2801_25 | + | + | + | + | Nygren et al. study | COI: MG025345  16S: MG025509  28S: MG024882  ITS: MG025435 |
| 2809_25 | + | + | + | + | Nygren et al. study | COI: MG025347  16S: MG025510  28S: MG024884  ITS: MG025436 |
| 2805_26 | + | + | + | + | Nygren et al. study | COI: MG025349  16S: MG025511  28S: MG024885  ITS: MG025437 |
| 2807_26 | - | + | + | + | Nygren et al. study | 16S: MG025512  28S: MG024886  ITS: MG025438 |
| 2800_27 | + | + | + | + | Nygren et al. study | COI: MG025350  16S: MG025513  28S: MG024888  ITS: MG025439 |
| 2324_28 | + | + | + | + | Nygren et al. study | COI: MG025351  16S: MG025514  28S: MG024889  ITS: MG025440 |
| 2875_28 | + | + | + | + | Nygren et al. study | COI: MG025354  16S: MG025516  28S: MG024892  ITS: MG025441 |

*These names describe the color of the live specimens, but the color apparently is not a morphological feature, but could be the result of the way of life.

** Repository numbers in the museum of Shirshov Institute of Oceanology, Russian Academy of Sciences.

**Table S2.** Best-fit models for datasets determined using the Akaike information criteria (AICc)

| Dataset | Model | |
| --- | --- | --- |
| COI | GTR + F+ I + G4 (1^st^ and 2^nd^)  GTR + I + G4  (3^rd^) | General Time Reversible model with a proportion of the sites invariable and gamma distributed rates across sites |
| 16S | GTR + I + G4 | General Time Reversible model with a proportion of the sites invariable and gamma distributed rates across sites |
| 28S | TIM3* + I + G4 | General Time Reversible model with a proportion of the sites invariable and gamma distributed rates across sites |
| ITS | TN* + I + G4 | General Time Reversible model with a proportion of the sites invariable and gamma distributed rates across sites |
| Four concatenated genes | SYM + I + G | Symmetric model with unequal rates but equal base freq with a proportion of the sites invariable and gamma distributed rates across sites |

*Were replaced by GTR


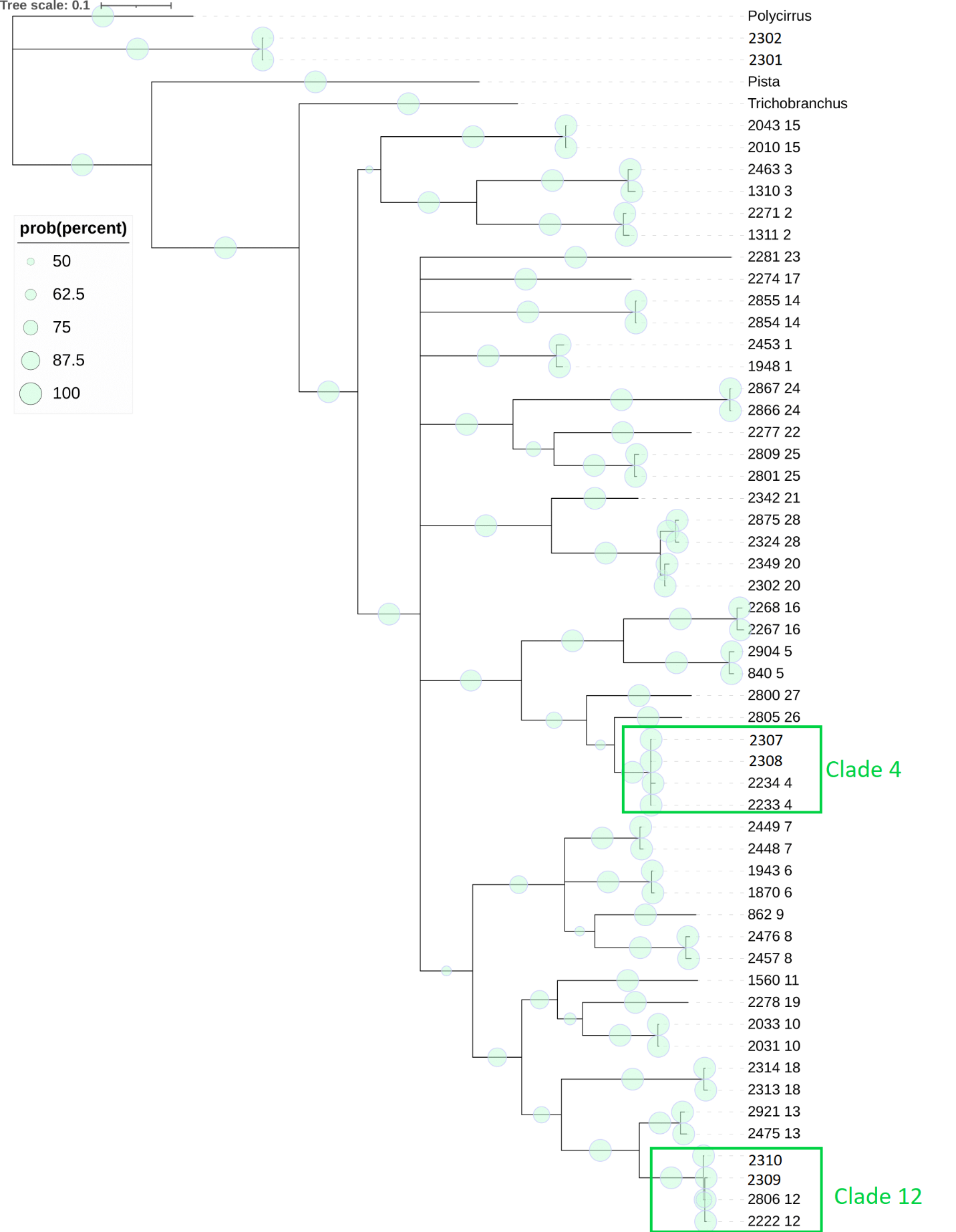


**Figure S1.** Phylogenetic tree, according to the analysis of 658 bp sequences from the COI gene fragment. The phylogenetic tree was constructed using the Bayesian analysis (BA). Clade 4 and Clade 12 are new undescribed species according to the study Nygren and colleagues (2018). Bayesian posterior probabilities (in percent) are shown as circles.

**
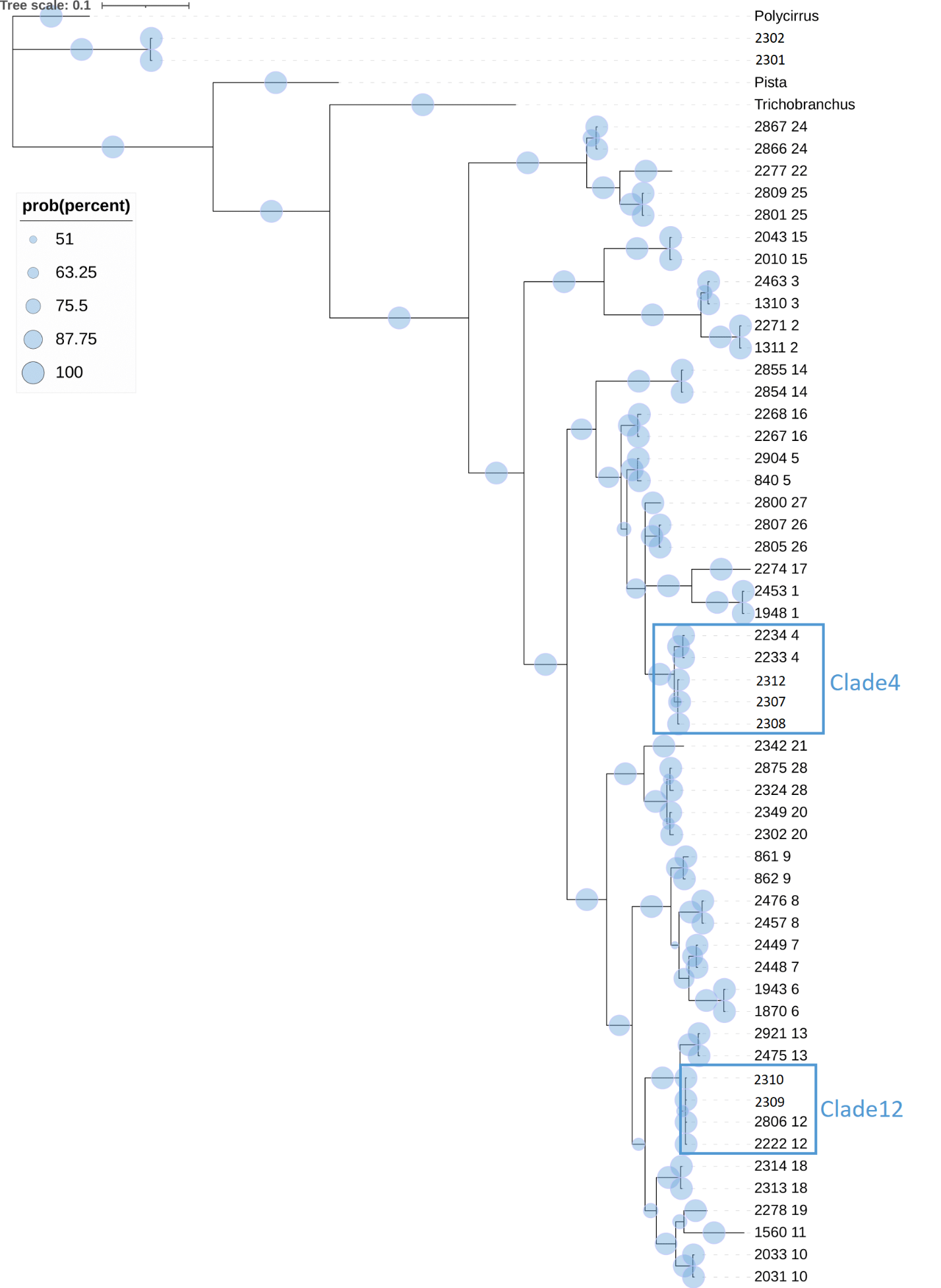
**

**Figure S2.** Phylogenetic tree, according to the analysis of 464 bp sequences from the 16S gene fragment. The phylogenetic tree was constructed using the Bayesian analysis (BA). Clade 4 and Clade 12 are new undescribed species according to the study Nygren and colleagues (2018). Bayesian posterior probabilities (in percent) are shown as circles.


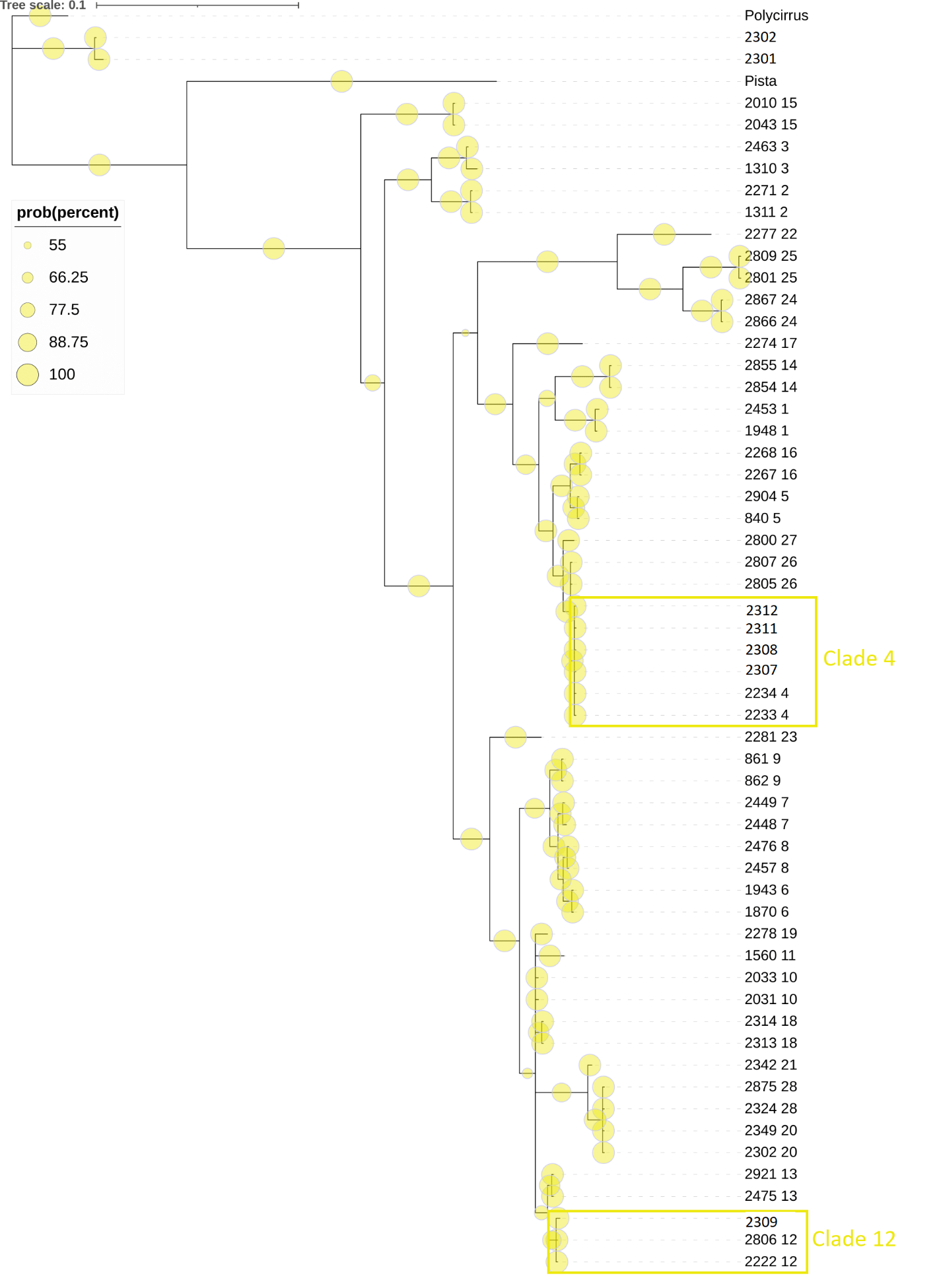


**Figure S3.** Phylogenetic tree, according to the analysis of approximately 842 bp sequences from the 28S gene fragment. The phylogenetic tree was constructed using the Bayesian analysis (BA). Clade 4 and Clade 12 are new undescribed species according to the study Nygren and colleagues (2018). Bayesian posterior probabilities (in percent) are shown as circles.

**
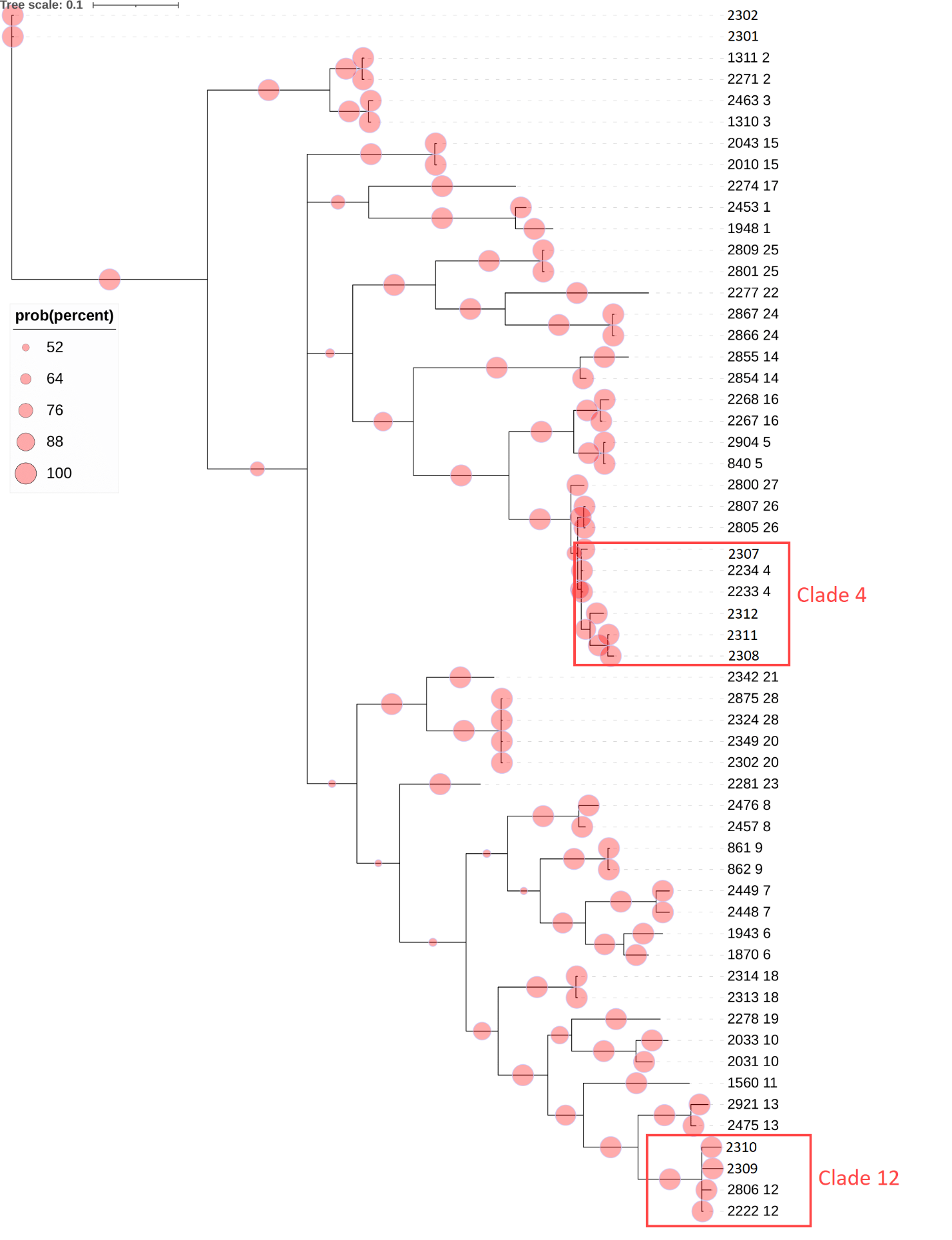
**

**Figure S4.** Phylogenetic tree, according to the analysis of 481bp sequences from the ITS2 gene fragment. The phylogenetic tree was constructed using the Bayesian analysis (BA). Clade 4 and Clade 12 are new undescribed species according to the study Nygren and colleagues (2018). Bayesian posterior probabilities (in percent) are shown as circles.

**Table S3.** Uncorrected distances between the neighboring clades 4, 26 and 27. Intraspecific distance inside Сlade 4 (including 2307, 2308, 2311 and 2312) is shown in bold.

|  | Clade 4 |
| --- | --- |
| Clade 4 | **0,0067** |
| Clade 26 | 0,0278 |
| Clade 27 | 0,0481 |

**Table S4.** Number of studied specimens.

|  | All depths | | | 10-25m depths | | 10-170m depths | |
| --- | --- | --- | --- | --- | --- | --- | --- |
|  | All specimens | Clade 4 specimens | Clade 12 specimens | All specimens | Clade 4 specimens | All specimens | Clade 12 specimens |
| Norwegian coast and shelf | 192 | 0 | 0 | 6 | 0 | 60 | 0 |
| Kattegat | 25 | 13 | 5 | 18 | 13 | 25 | 5 |
| Skagerrak | 108 | 1 | 14 | 7 | 1 | 83 | 14 |
| White Sea | 6 | 4 | 2 | 6 | 4 | 6 | 2 |
| Barents Sea | 100 | 0 | 17 | 0 | 0 | 37 | 17 |

**Table S5.** The results of the one-tailed t-test to establish the statistical significance of the difference between the Clade 4 density distribution along the Norwegian coast and the each of three locations (Kattegat, Skagerrak, White Sea). P-values are shown.

|  | p-value (All depths) | p-value (10-25m depths) |
| --- | --- | --- |
| Kattegat | <0.00001 | 0.000513 |
| Skagerrak | 0.091435 | 0.188687 |
| White Sea | <0.00001 | 0.00506 |

**Table S6.** The results of the one-tailed t-test to establish the statistical significance of the difference between the Clade 12 density distribution along the Norwegian coast and the each of four locations (Kattegat, Skagerrak, White Sea, Barents Sea). P-values are shown.

|  | p-value (All depths) | p-value (10-170m depths) |
| --- | --- | --- |
| Kattegat | <0.00001 | 0.000125 |
| Skagerrak | <0.00001 | 0.000352 |
| White Sea | <0.00001 | <0.00001 |
| Barents Sea | <0.00001 | <0.00001 |
